# Application of the Neuroform Atlas Stent in Intracranial Aneurysms: Current Status

**DOI:** 10.3389/fneur.2022.829143

**Published:** 2022-03-25

**Authors:** Kun Hou, Jinlu Yu

**Affiliations:** Department of Neurosurgery, First Hospital of Jilin University, Changchun, China

**Keywords:** Neuroform Atlas stent, aneurysm, endovascular treatment, review, prognosis

## Abstract

The Neuroform Atlas stent (NAS) is the successor of the Neuroform EZ stent. The NAS is compatible with a low-profile 0.0165-inch microcatheter and is soft enough to pass through small and highly tortuous vessels. The NAS can be used in treating intracranial aneurysms at almost all locations, and its use is becoming increasingly common. However, there has not yet been a complete review of NAS applications. Therefore, we performed this review, which addresses several aspects of the NAS, mainly including its characteristics, clinical trials of its application in treating aneurysms, deployment techniques for the device, the prognosis and complications of its application in treating aneurysms, and antiplatelet requirements associated with its use. Based on the evidence reviewed here, as well as our experience, we found that the NAS is a promising device for treating intracranial aneurysms, especially complex and distal aneurysms. This stent can also be used as a powerful tool to assist in rescuing coil migration, completing dual-stent reconstruction, and coiling aneurysms *via* a transcirculation approach. The device may require antiplatelet therapy at a lower dose and over a shorter period than other stents. The deployment of the NAS to assist in aneurysm coiling can yield good clinical outcomes and an acceptable rate of complications. Thus, the NAS is a promising device.

## Introduction

**Figure 1:**
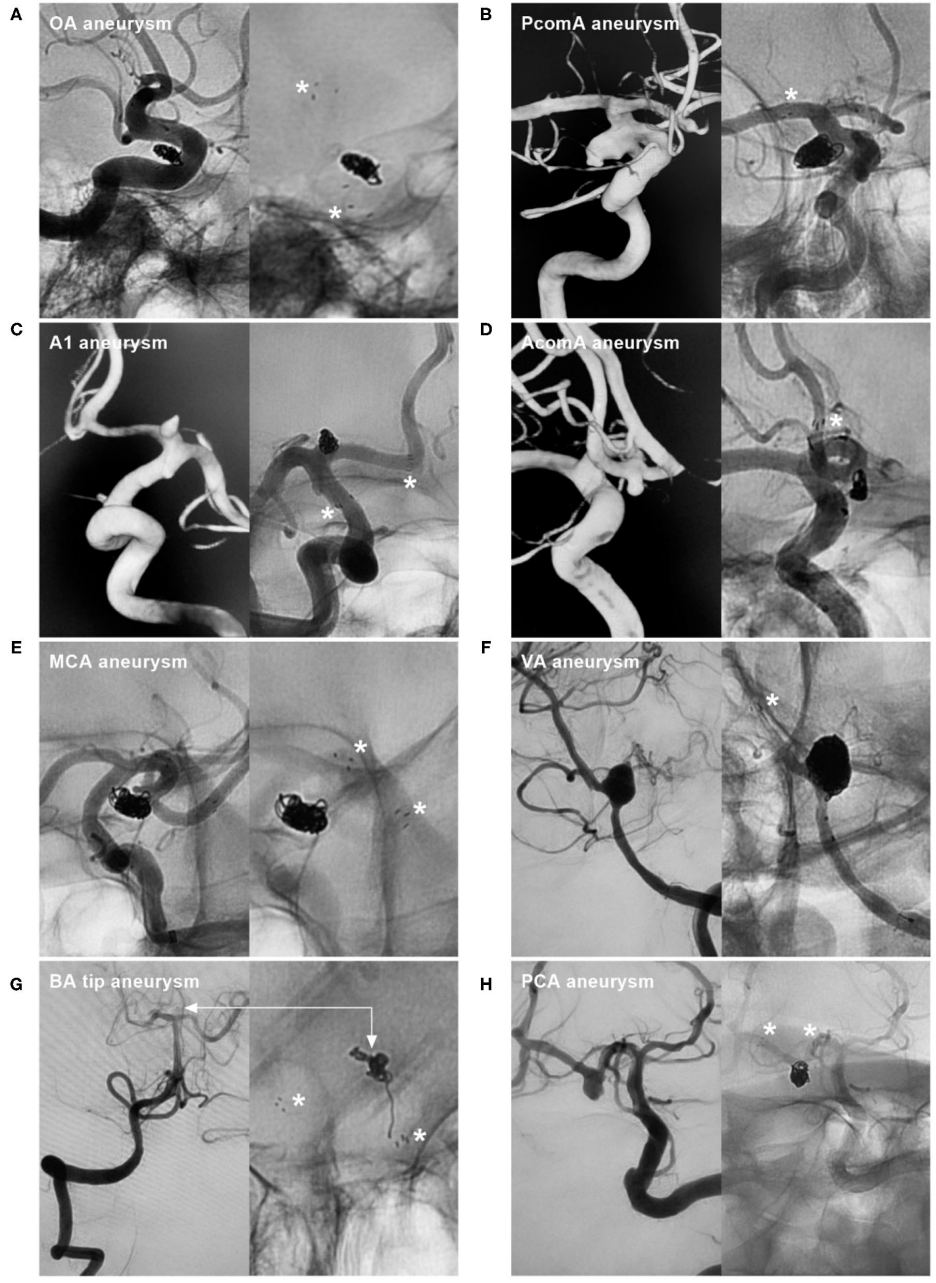
NAS application to treat aneurysms at various locations. **(A)** NAS-assisted coiling of an OA aneurysm; the asterisks in the right image indicate proximal and distal markers. **(B)** NAS-assisted coiling of a PcomA aneurysm; the asterisk in the right image indicates the distal marker. **(C)** NAS-assisted coiling of an A1 aneurysm; the asterisks in the right image indicate the proximal and distal markers. **(D)** NAS-assisted coiling of an AcomA aneurysm; the asterisk in the right image indicates the distal marker. **(E)** NAS-assisted coiling of an MCA aneurysm; the asterisks in the right image indicate the proximal and distal markers. **(F)** NAS-assisted coiling of a VA aneurysm; the asterisk in the right image indicates the distal marker. **(G)** NAS-assisted coiling of a basilar tip aneurysm; the asterisks in the right image indicate the proximal and distal markers, and the bidirectional arrows indicate the aneurysm location. **(H)** NAS-assisted coiling of a PCA aneurysm; the asterisks in the right image indicate the proximal and distal markers. A1, First segment of the anterior cerebral artery; AcomA, anterior communicating artery; BA, basilar artery; MCA, middle cerebral artery; NAS, Neuroform Atlas stent; OA, ophthalmic artery; PCA, posterior cerebral artery; PcomA, posterior communicating artery; VA, vertebral artery.

The Neuroform Atlas stent (NAS) (Stryker Neurovascular, Fremont, California, USA) is a self-expanding nitinol stent that represents a recent advance in intracranial stents for endovascular treatment (EVT). It can be used as a scaffold to support intrasaccular coils and assist in aneurysm embolization (1). This NAS received US Food and Drug Administration (FDA) approval on 16 May 2019 (2). Currently, the use of the NAS to assist aneurysm coiling is becoming increasingly popular. The NAS can be used to treat aneurysms at almost all locations (Figure 1). Although experience with the NAS in treating intracranial aneurysms is accumulating, there has not yet been a comprehensive review of the current applications of this device. Therefore, we reviewed data from the PubMed database (last date, 2022-2-15) and information from our own clinical experience, which we summarize herein. Additionally, we present some educational and illustrative cases.

## Characteristics of the NAS

The NAS uses a hybrid-cell design; it has an open-cell structure through most of its length, with a closed-cell structure at its proximal end, which serves to increase device stability upon recrossing with a microcatheter. The open-cell structure maintains the lumen and conforms to the vessel wall, achieving good apposition (3). The NAS is the successor of the Neuroform EZ stent (Stryker, Kalamazoo, MI, USA). Owing to reduced strut thickness and a reduction in the number of radiopaque markers from four in the Neuroform EZ stent to three in the NAS, the NAS can pass through low-profile 0.0165-inch microcatheters [Excelsior SL-10^®^ and XT-17™ (Stryker Neurovascular, Fremont, California, USA), Echelon-10 (Medtronic, Irvine, California, USA), etc.] (4). Therefore, it is soft enough to pass through small and highly tortuous vessels. The NAS is intended for use in vessels from 2.0 to 4.5 mm in diameter (5, 6).

Reducing the metal volume weakens the visualization of the NAS during deployment; moreover, the proximal segment is less stable due to the closed-cell design. Slight foreshortening (2.9–6.3 mm, depending on the vessel size) should be expected to occur after NAS deployment (7). Owing to its reduced metal content, the NAS has a low rate of flow diversion. Simultaneously, however, the deployment of a braided stent in an NAS can lead to increased metal coverage of the aneurysm neck and a shortened transition zone, which may contribute to aneurysm occlusion by increasing the flow-diverting effect (8).

## Clinical Trials of the NAS in Aneurysm Treatment

Clinical trials of the NAS have been performed with the aim of using it to assist in the coiling of wide-necked intracranial aneurysms, which were defined by a neck ≥4 mm and a dome-to-neck ratio <2 (5, 6, 9–11). Recently, three prospective trials (2020–2022) with large series have been performed (9–11).

The first was a trial of NAS-assisted coiling of anterior circulation aneurysms; in total, 182 patients were enrolled. The technical success rate was 100%, the aneurysm size was 6.1 ± 2.2 mm, and adequate occlusion [Raymond-Roy (12) (RR) class 1 or 2] was observed in 96.1% of patients on follow-up (9). The second was a trial of NAS-assisted coiling of posterior circulation aneurysms; in total, 116 patients were enrolled. The technical success rate was 100%, the aneurysm size was 7.1 ± 3.0 mm, and adequate occlusion was observed in 94.7% of patients on follow-up (11). The third was a multicentric European post-market follow-up study of the NAS-assisted coiling of anterior (90.5%) and posterior (9.5%) circulation aneurysms; in all, 105 patients were enrolled. The technical success rate was 94.7%, the mean aneurysm size was 5.8 ± 2.5 mm, and adequate occlusion was found in 98.9% of patients on follow-up (10). In these trials, the prognosis after EVT was satisfactory, with adequate occlusion in approximately 95% of patients (9–11).

Currently, intracranial aneurysms are categorized as small (<7 mm), medium (7–12 mm), large (>12–25 mm) or giant (>25 mm) (13). In the three trials mentioned above, most aneurysms in the anterior and posterior circulation were small and medium, respectively, indicating that NAS-assisted coiling is effective for small and medium aneurysms (9–11).

In addition, two systematic reviews and meta-analyses of the NAS for aneurysm treatment have been performed. Pranata et al. reviewed published data (2018–2019) on 568 intracranial aneurysms in 557 patients and found an adequate occlusion rate of 93% at the end of follow-up (4). Lynch et al. reviewed published data (2018–2020) encompassing 593 intracranial aneurysms in 577 patients and found an adequate occlusion rate of 94.8% (14). The results of these two studies are similar to those of the three abovementioned trials (9–11).

**Figure 2:**
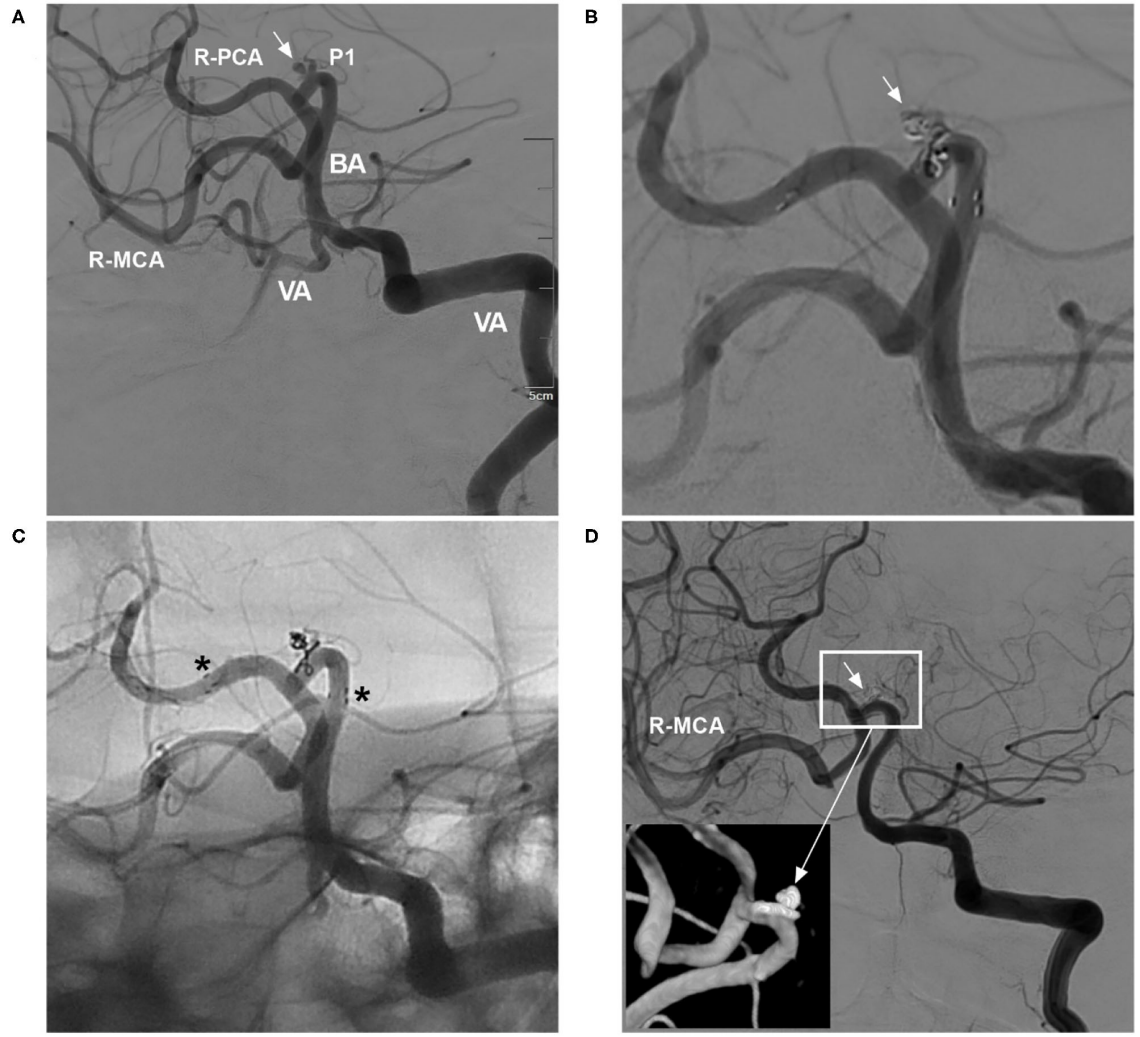
Delayed aneurysm occlusion. **(A)** DSA of the VA showing an aneurysm (arrow) in the right P1 segment; the right ICA was occluded, and the right MCA was supplied by the posterior circulation *via* the posterior communicating artery. **(B)** DSA of the VA showing incomplete embolization with Raymond-Roy class 3 occlusion of the aneurysm (arrow). **(C)** Unsubtracted DSA showing that the NAS pushed the herniated coils against the vessel wall; the asterisks show the proximal and distal markers. **(D)** Follow-up DSA of the VA showing complete occlusion (Raymond-Roy class 1) of the aneurysm (short arrow); the magnified inset (long arrow from the frame) shows aneurysm coiling. BA, basilar artery; DSA, digital subtraction angiography; ICA, internal carotid artery; MCA, middle carotid artery; NAS, Neuroform Atlas stent; P1, first segment of the PCA; PCA, posterior carotid artery; R, right; VA, vertebral artery.

In three clinical trials and two meta-analyses of the NAS for treating aneurysms, the final aneurysm occlusion rate was higher than the immediate occlusion rate due to progressive thrombosis, which tended to occur in small aneurysms (4, 9–11, 14). Therefore, for small aneurysms, even if immediate complete occlusion is not achieved after EVT, the result on follow-up can still be good (Figure 2).

In addition, some other retrospective studies of NAS-assisted coiling of aneurysms and studies comparing NAS with other intracranial stents also revealed excellent results after NAS-assisted coiling of aneurysms (15–17). Recently, NAS-assisted coiling of ruptured intracranial aneurysms has become a feasible option when simple coiling is not possible (18).

## Techniques for NAS Deployment

### Jailing and Transcell Techniques

**Figure 3:**
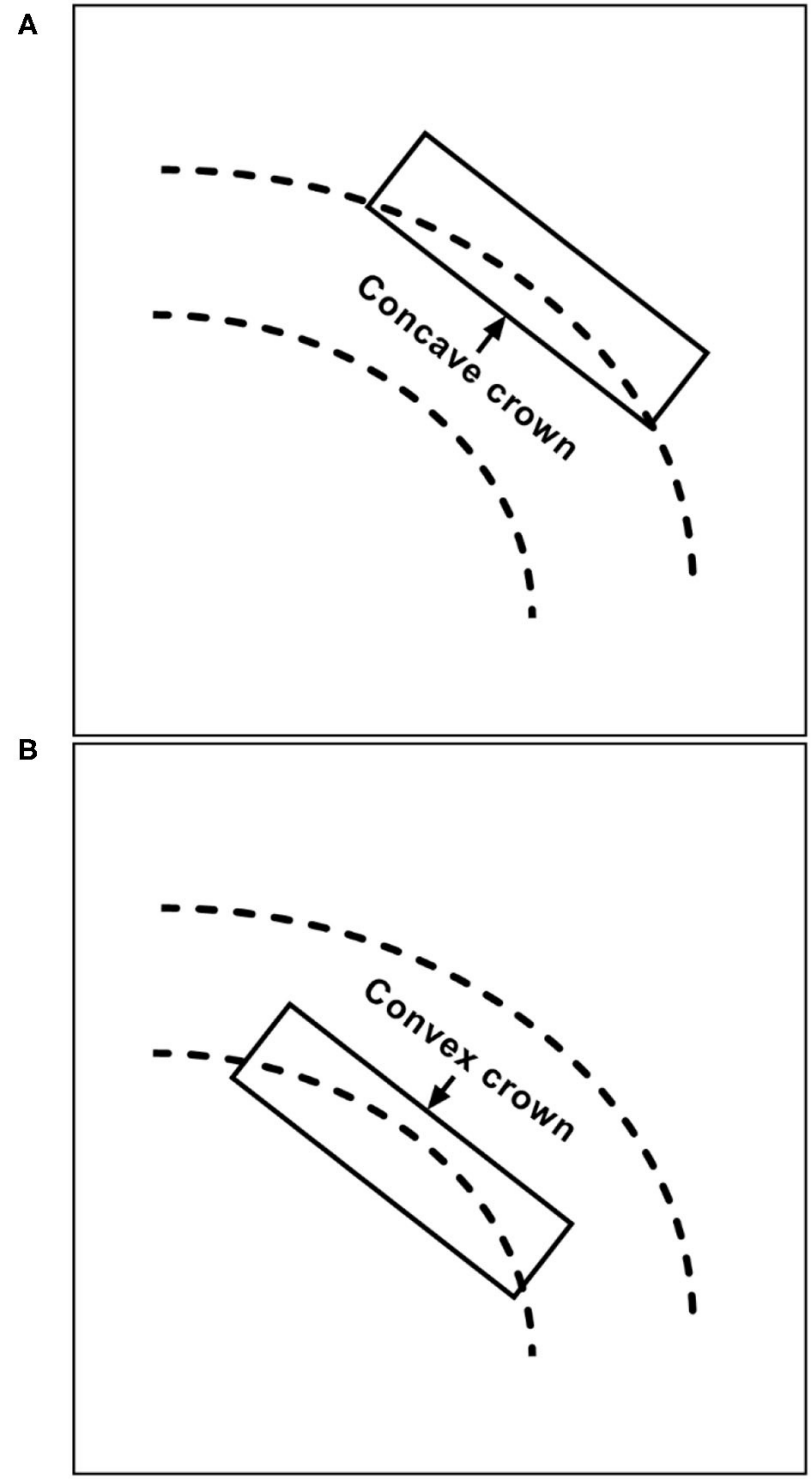
Illustration of **(B)** convex and **(A)** concave crowns in the structure of the NAS. NAS, Neuroform Atlas stent.

After the microcatheter for coil delivery is positioned, the stent can be released or partially released to assist in coiling; these techniques are known as jailing and semi-jailing, respectively (19, 20). The jailing technique restricts the movement of the coil delivery microcatheter, resulting in difficulties achieving paint-brushing or back-and-forth movement of the microcatheter (21). In the transcell technique, although the microcatheter can move freely, it can easily be forced out of the aneurysm during coiling. In addition, the transcell technique is difficult because the microcatheter might become stuck in the struts of the NAS. Certain parts of the NAS are easier to cross than others; in a study by Hanaoka et al., it was easier to cross the concave crown than the convex crown (Figure 3) (22).

**Figure 4:**
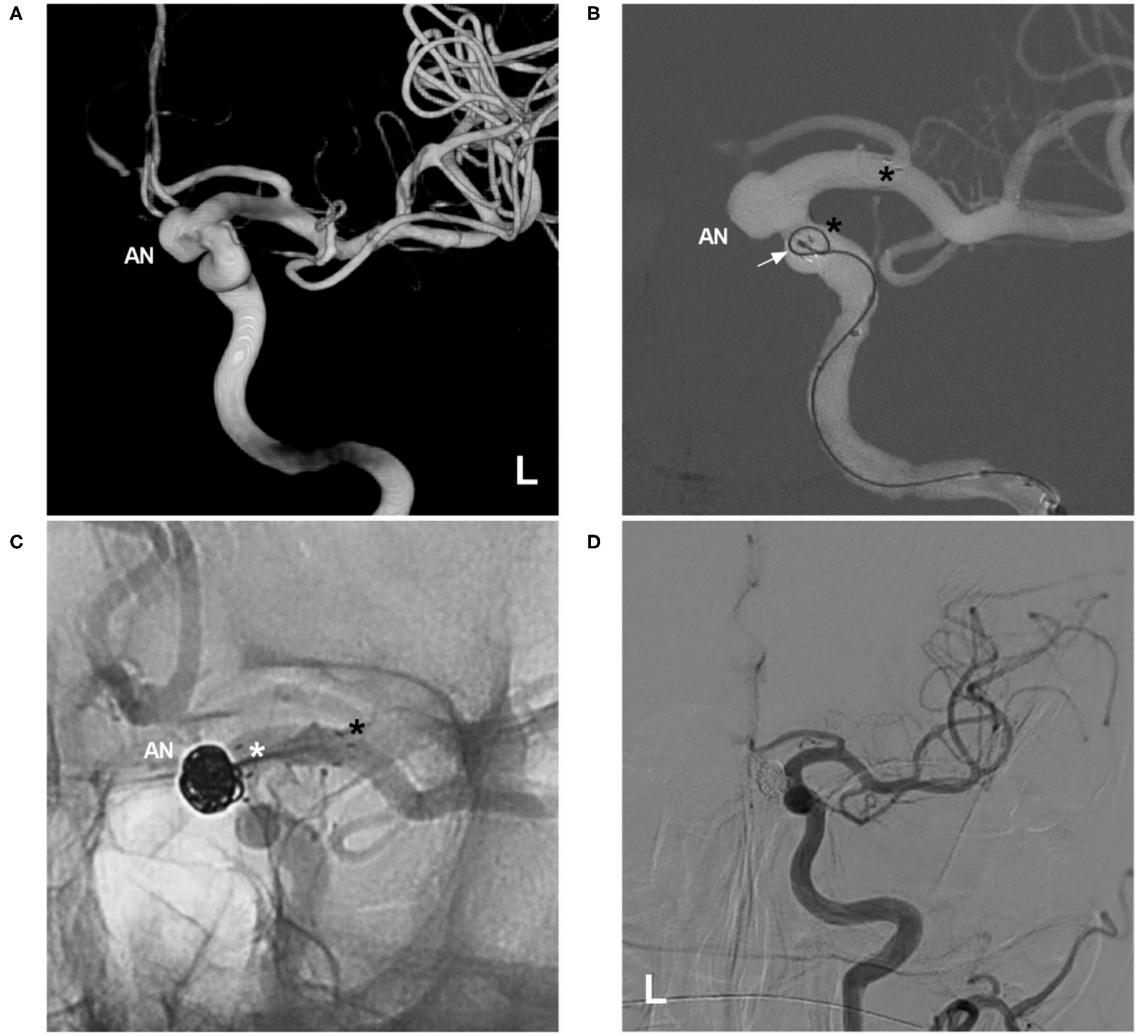
Migration of the proximal NAS segment during the transcell procedure. **(A)** Three-dimensional DSA of the left ICA showing an ophthalmic artery aneurysm (AN). **(B)** Map showing deployment of the NAS first; the asterisks indicate the proximal and distal tips, and the arrow indicates the microcatheter during an attempt to cross the NAS. **(C)** Unsubtracted DSA showing that the proximal segment of the NAS (white asterisk) was pushed distally by the microcatheter tip, part of the aneurysm neck was uncovered, the distal segment of the stent (black asterisk) was not moved, and the aneurysm (AN) was still coiled under incomplete protection by the NAS. **(D)** Follow-up DSA of the left ICA showing complete embolization of the aneurysm. AN, aneurysm; DSA, digital subtraction angiography; ICA, internal carotid artery; NAS, Neuroform Atlas stent; L, left.

Therefore, the jailing and transcell techniques should be evaluated as options on a case-by-case basis. In a multicentric European follow-up study, the jailing technique was used in 60.9% of cases, and the transcell technique was used in 32.4% of cases (10). Sometimes, the transcell technique is the last resort in the treatment of wide-necked aneurysms in a parent artery with a small caliber, stenosis, or a tortuous course because double microcatheters have difficulty passing through the parent artery (23, 24). Additionally, the transcell technique may be dangerous because the microcatheter can suddenly jump into the aneurysm sac, perforating the aneurysm. Because the closed-cell proximal segment of the NAS is relatively unstable, this segment can be pushed distally by the microcatheter tip during the transcell procedure (Figure 4). A J-shaped microguidewire can be helpful to avoid NAS migration.

### Dual-Stent Techniques

**Figure 5:**
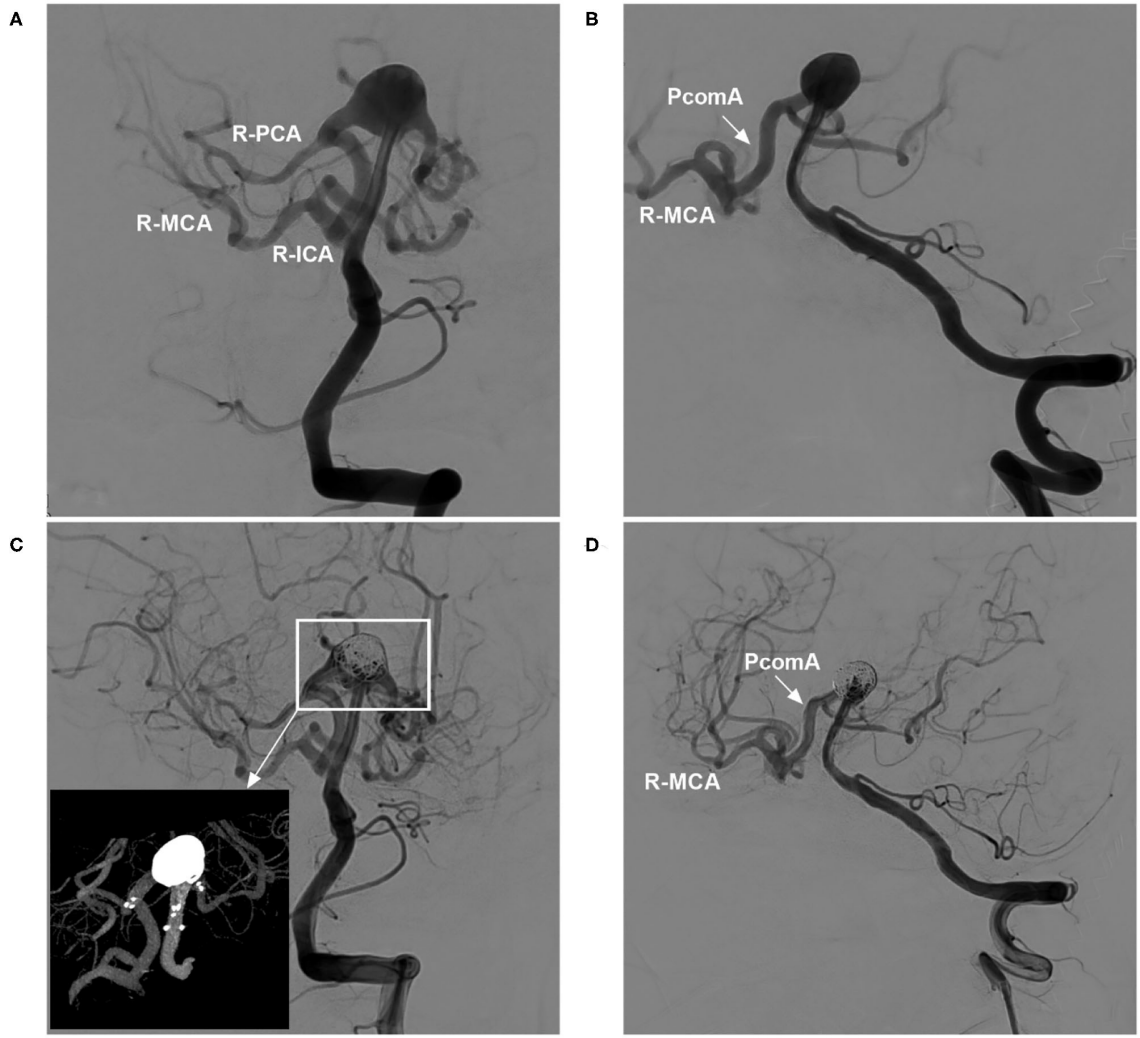
Cross Y-stent-assisted coiling of a basilar apex aneurysm in ICA occlusion. **(A,B)** DSA of the VA showing a basilar apex aneurysm, with the right ICA system supplied *via* the PcomA (arrow in **B**) from the posterior circulation; **(A)** presents an approximately anteroposterior view, while B presents an oblique view. **(C)** DSA of the VA showing coiling of the aneurysm with the assistance of cross Y-stenting; the magnified inset (long arrow from the frame) shows the NAS and coiling. **(D)** DSA of the VA (oblique view) showing that the arteries around the coiled aneurysm were not affected and that the PcomA (arrow) supplied the right ICA system. DSA, digital subtraction angiography; ICA, internal carotid artery; MCA, middle cerebral artery; NAS, Neuroform Atlas stent; PCA, posterior cerebral artery; PcomA, posterior communicating artery; R, right; VA, vertebral artery.

Dual-stent techniques include Y- and X-stenting, with Y-stenting being more common (25–28). They are effective for treating complex bifurcation aneurysms, which are defined as wide-necked aneurysms that incorporate more than one branch (26, 27, 29, 30). Previously, dual-stent techniques could be performed only through 0.021- or 0.027-inch microcatheters and were often difficult to realize (29). The NAS is compatible with 0.0165-inch microcatheters, and the open-cell structure promotes sufficient expansion of the second NAS at the intersection point (15). Therefore, the NAS has the advantage of being suitable for use in dual stenting (Figure 5).

During Y-stenting, the first NAS should be deployed in the most challenging branch (31). The “around the world” technique is helpful in large or giant aneurysms, as the microguidewire can be deflected off the aneurysm dome and turned into the branch. Then, the microguidewire loop is taken up by withdrawing the wire, and the microcatheter is positioned along the microguidewire. Later, the microcatheter crosses the first NAS to complete the catheterization and Y-stenting (32). After Y-stenting, a microcatheter needs to cross the NAS to coil the aneurysm. To avoid transcell difficulty, the microcatheter may be jailed in the aneurysm before the first NAS deployment.

Dual-NAS-assisted aneurysm coiling is effective. In a report by Aydin et al. of 30 aneurysms treated with NAS Y-stenting, the rate of adequate occlusion on follow-up was 93.3% (29). In Ciccio et al.'s report of 55 aneurysms treated with NAS Y- and X-stenting, the rate of adequate occlusion on follow-up was 95% (26). These results of dual-NAS-assisted aneurysm coiling are similar to those of recent meta-analyses. For example, in a meta-analysis by Cagnazzo et al. of Y-stent-assisted aneurysm coiling, the rate of adequate occlusion on follow-up was 95.4% (33). Additionally, a meta-analysis by Granja et al. of Y-stent-assisted aneurysm coiling, the rate was 91% (34).

Based on the stent construction, Y-stenting can be subdivided into the crossing and kissing types (35). When the proximal parent artery has a diameter <4 mm, crossing stenting is performed. When the proximal parent artery has a diameter ≥4 mm, kissing stents can be arranged. Sato et al. reported no significant differences between crossing and kissing Y-stenting (25). In addition to preventing coil protrusion, Y-stenting can lead to immediate as well as delayed angular remodeling of both affected branches to prevent aneurysm recurrence (36).

### Transcirculation and Antegrade Horizontal Stenting

#### Transcirculation Stenting

Transcirculation stenting is defined as accessing two of the four intracranial circulations (right anterior, left anterior, right posterior, and left posterior) to perform coiling from one circulation and stenting from another (37). Transcirculation stenting involves the horizontal deployment of a stent across the aneurysm neck, and it is appropriate for treating certain wide-necked bifurcation aneurysms as well as those with acute-angle efferent branches (37–43). Aneurysms in the anterior circulation, such as aneurysms of the carotid terminus, A1, and posterior communicating artery (PcomA), can be accessed from the contralateral carotid artery *via* the anterior communicating artery (AcomA) and PcomA (38, 39, 44).

**Figure 6:**
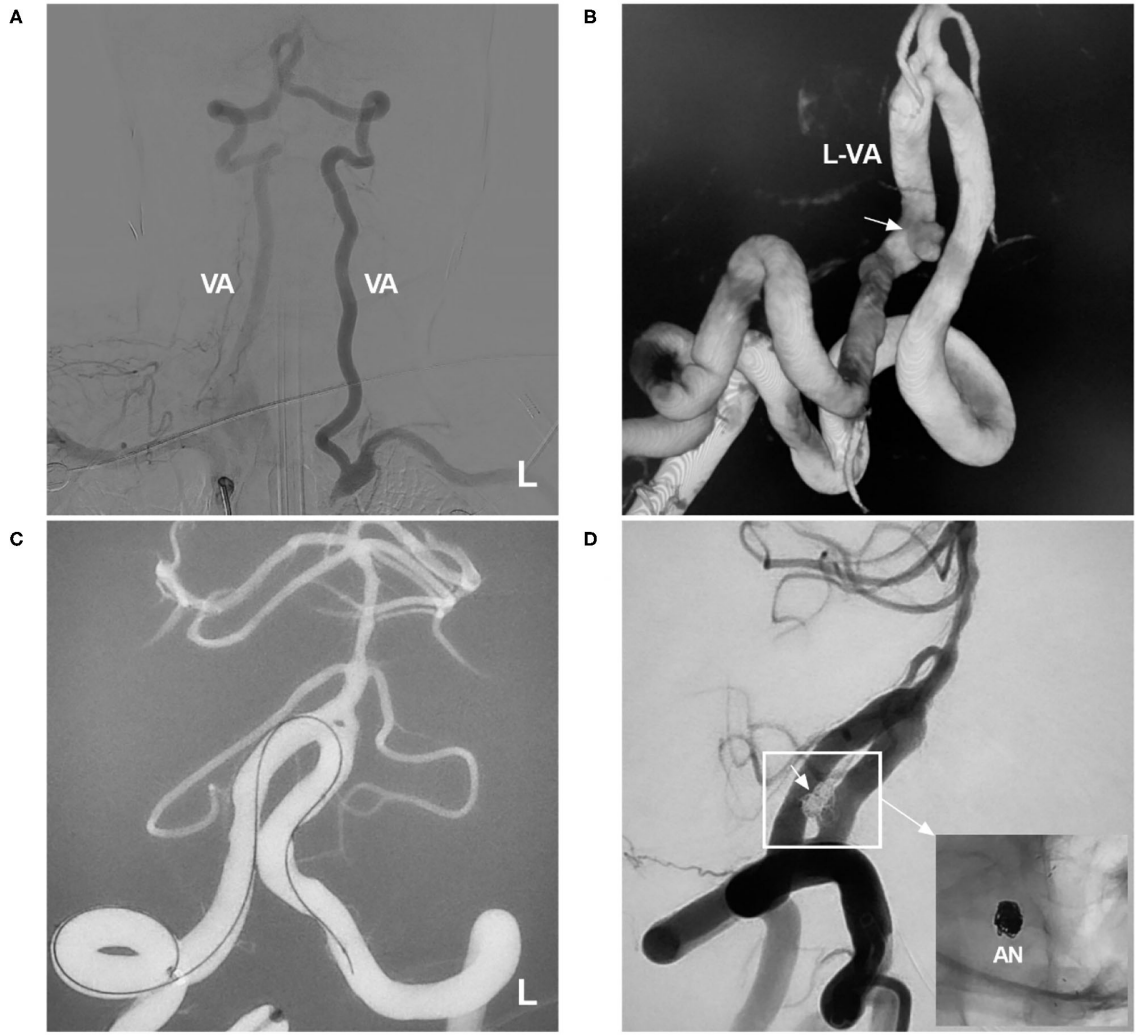
Transcirculation NAS-assisted coiling of a VA aneurysm. **(A)** DSA of the right subclavian artery showing occlusion of the left subclavian artery, with the blood-stealing phenomenon from the VA to the subclavian artery. **(B)** Three-dimensional DSA of the VA showing the aneurysm (arrow) in the left VA. **(C)** NAS delivery microcatheter advances into the contralateral VA under the navigation of roadmap. **(D)** Postoperative DSA of the VA showing complete occlusion of the aneurysm (arrow); the magnified inset (long arrow from the frame) shows NAS-assisted coiling of the aneurysm (AN). AN, aneurysm; DSA, digital subtraction angiography; L, left; NAS, Neuroform Atlas stent; VA, vertebral artery.

Among aneurysms in the posterior circulation, basilar apex aneurysms can be accessed from the carotid artery *via* the PcomA. In posterior inferior cerebellar artery (PICA) aneurysms, when the proximal vertebral artery (VA) and PICA form an acute angle, stenting can be achieved by navigating through the contralateral VA into the PICA in conjunction with antegrade coiling of the aneurysm (45, 46). In addition, when the VA is occluded due to occlusion of the subclavian artery, transcirculation stenting can occasionally serve as a last resort to repair a dissecting aneurysm in the VA (Figure 6).

Previously, transcirculation stenting could be performed only with an Enterprise (Codman Neurovascular, Raynham, MA) or Neuroform EZ stent (38–40). A communicating artery with a diameter of 1.5 mm was necessary to allow the passage of an Enterprise stent delivery microcatheter, and a diameter of 2 mm was required for a Neuroform stent delivery microcatheter (38, 39). Catheterization using these 0.021- or 0.027-inch stent delivery microcatheters is difficult and may be associated with an increased risk of both thromboembolic and hemorrhagic complications (37, 45, 47).

**Figure 7:**
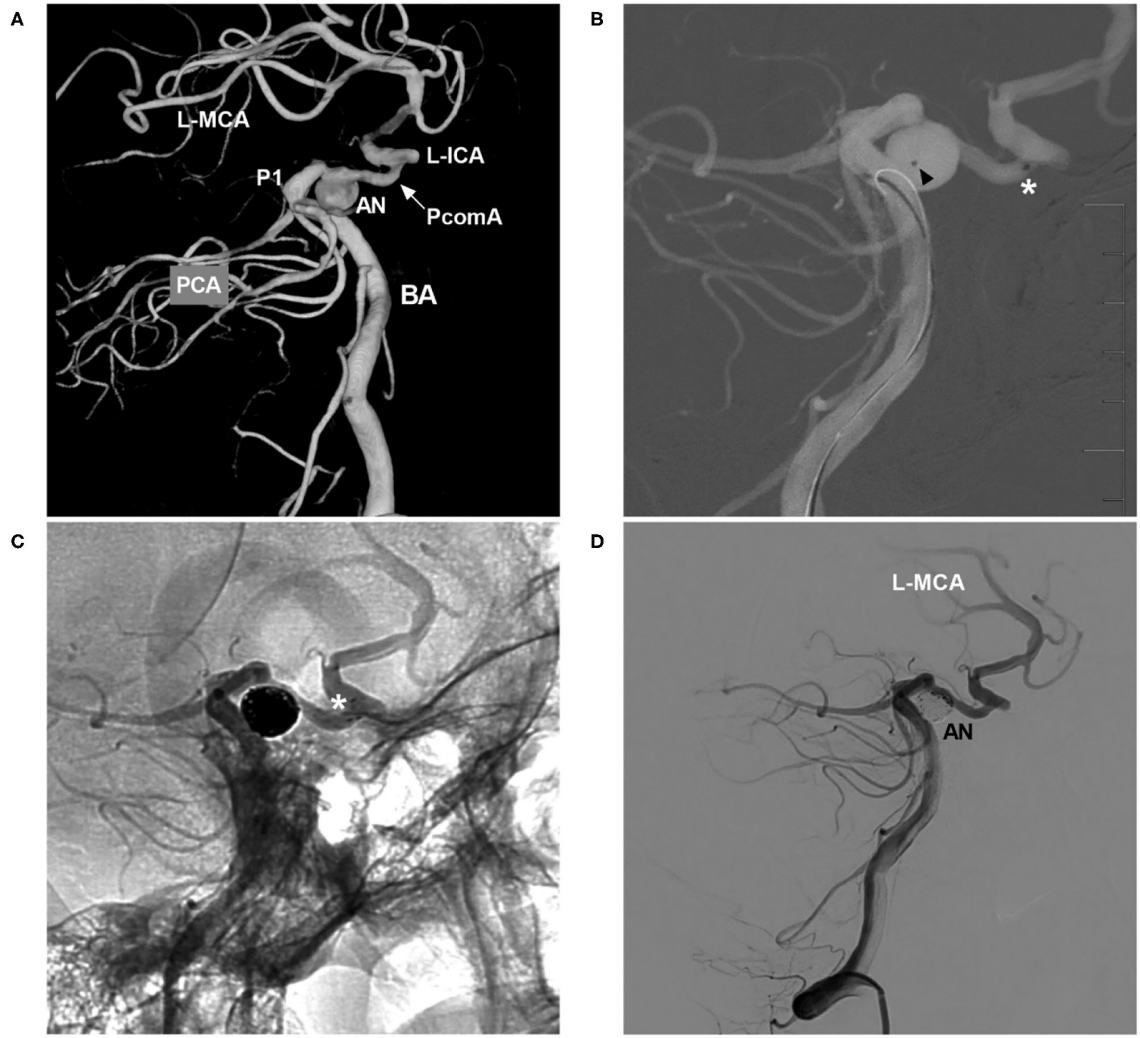
NAS-assisted coiling of a PcomA aneurysm with coil delivery from the posterior circulation to the anterior circulation. **(A)** Three-dimensional DSA of the BA showing occlusion of the left ICA, with the posterior circulation supplying the left MCA *via* the PcomA (arrow) and an aneurysm (AN) located at the left PcomA trunk near the PCA. **(B)** Map showing the positioning of the NAS delivery microcatheter (the asterisk indicates the distal marker) and the coil delivery microcatheter (the triangle indicates the tip) from the posterior circulation. **(C)** Unsubtracted DSA showing NAS deployment (the white asterisk shows the distal marker). **(D)** Postoperative DSA of the VA showing complete occlusion of the aneurysm (AN). AN, aneurysm; BA, basilar artery; DSA, digital subtraction angiography; ICA, internal carotid artery; L, left; MCA, middle carotid artery; NAS, Neuroform Atlas stent; P1, first segment of the PCA; PCA, posterior cerebral artery; PcomA, posterior communicating artery; VA, vertebral artery.

The NAS delivery microcatheter is soft and pliable, and it can pass through communicating arteries easily, which makes transcirculation stenting feasible. In Mascitelli et al.'s report of transcirculation stent-assisted aneurysm coiling, the NAS was used in 69% of patients, and the majority of patients (86.4%) had a good clinical outcome (37). During catheterization with the coil delivery microcatheter, the transcell technique through horizontal stents may be difficult. Therefore, jailing one or two coil delivery microcatheters can be helpful (40). In the case illustrated in Figure 7, the NAS was deployed from the posterior cerebral artery to the PcomA, and the jailing technique was used to complete aneurysm coiling.

#### Antegrade Horizontal Stenting

In this technique, stenting can be performed *via* an antegrade approach (48, 49). As a low-profile stent, the NAS is appropriate for this technique. First, the stent delivery microcatheter is steamed. Then, the curved part of the microcatheter is pre-positioned on the opposite side of the efferent vessel by looping. When the NAS is pushed, it can directly pass over the looped segment of the microcatheter, and antegrade NAS deployment is achieved by unsheathing the microcatheter (48). In China, this technique is called the “dragon swaying its tail” technique.

Antegrade horizontal stenting is not routine and is appropriate only for treating large middle cerebral artery and basilar apex aneurysms with sufficient space to loop the microcatheter. However, microcatheter and NAS delivery can be technically difficult due to the long course that must be navigated, and vessel perforation or dissection may occur during the procedure (48). A shorter NAS can be beneficial for tracking the looping curve.

**Figure 8:**
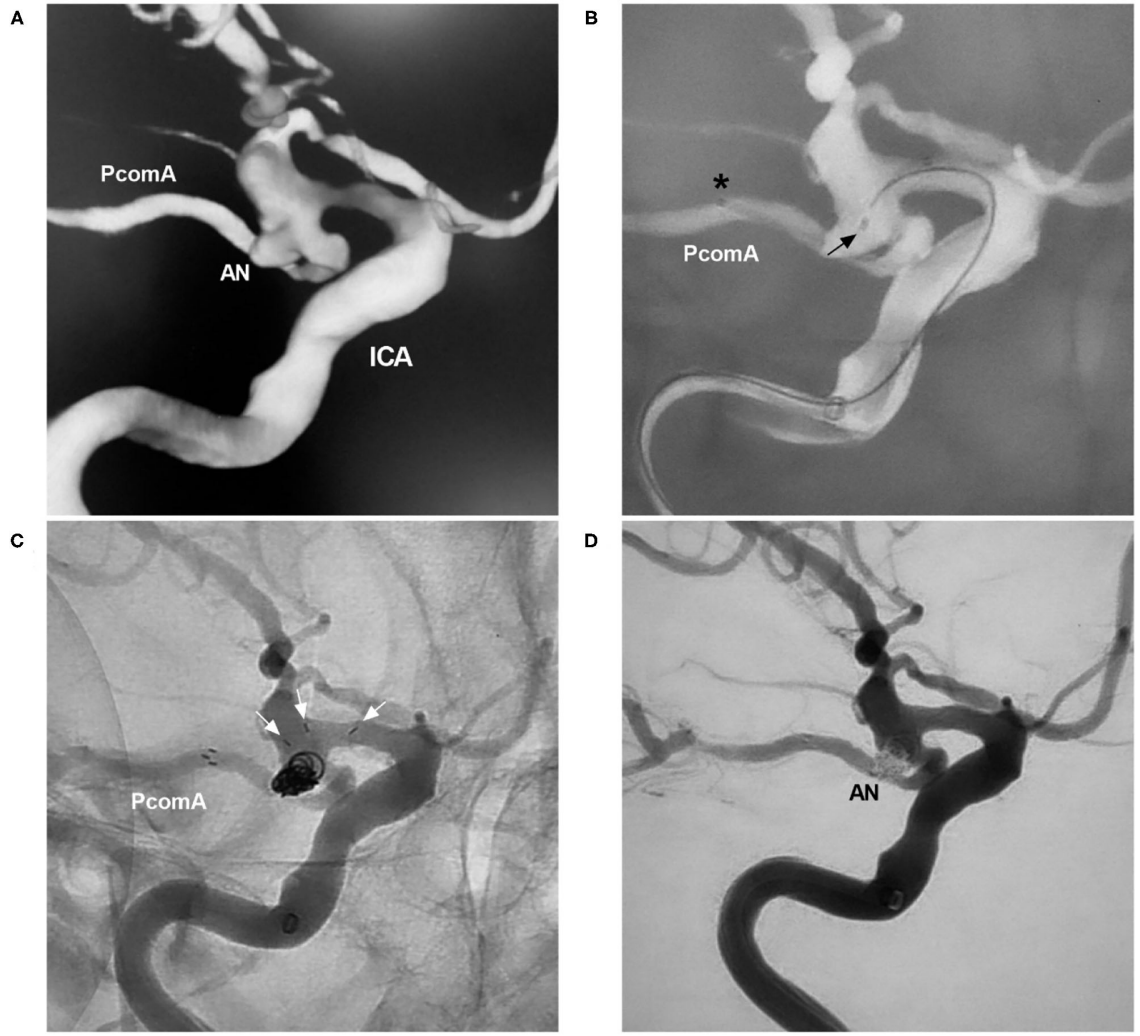
NAS-assisted coiling of a PcomA aneurysm. **(A)** Three-dimensional DSA of the ICA showing a PcomA aneurysm (AN). **(B)** Map showing the positioning of the NAS delivery microcatheter (the asterisk shows the distal marker) and the coil delivery microcatheter (the arrow shows the tip). **(C)** Unsubtracted DSA showing the opening of the proximal NAS segment (the arrows show three proximal markers) in the ICA. **(D)** Postoperative DSA of the ICA showing Raymond-Roy class 1 embolization of the aneurysm (AN). AN, aneurysm; DSA, digital subtraction angiography; ICA, internal carotid artery; PcomA, posterior communicating artery; NAS, Neuroform Atlas stent.

Traditional antegrade horizontal stenting is complex and difficult, and we have created a method to simplify it. In the case represented in Figure 8, the distal NAS was anchored first in the PcomA by applying some forward pressure to the delivery microcatheter during NAS deployment. The NAS was released such that the proximal stent markers abutted the wall of the internal carotid artery, with the stent markers at the ostium of the PcomA. However, in this technique, the exact landing point of the proximal NAS needs to be predicted.

### Herniation Technique

**Figure 9:**
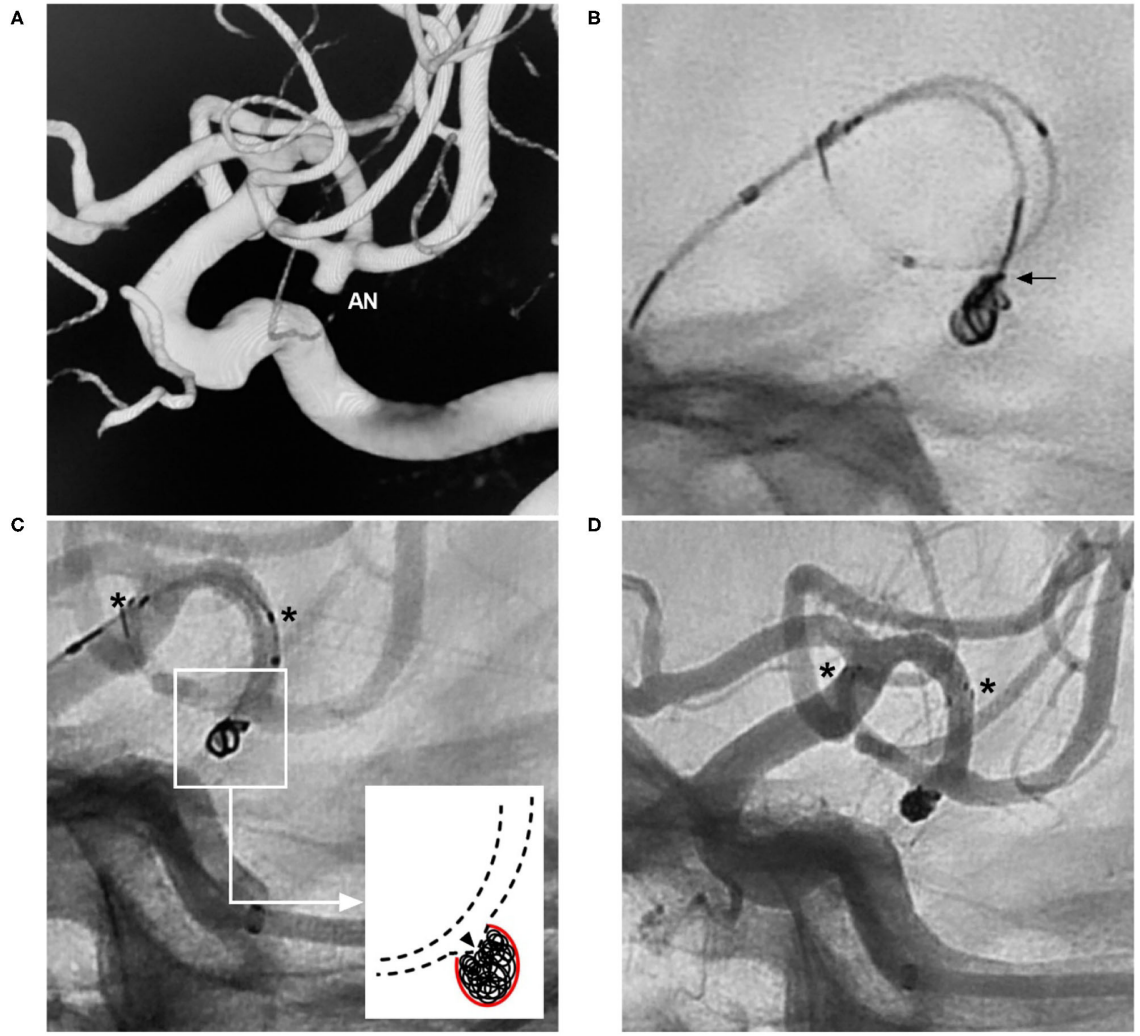
Herniation technique with the NAS. **(A)** Three-dimensional DSA of the ICA showing an MCA aneurysm (AN). **(B)** X-ray film showing protrusion of the coil into the parent artery (arrow). **(C)** Unsubtracted DSA showing that after NAS deployment, the coil was pushed into the aneurysm sac by the herniation technique; the asterisks show the proximal and distal markers of the NAS, and the magnified inset (long arrow from the frame) shows herniation of the struts (triangle) of the NAS into the aneurysm. **(D)** Postoperative DSA of the ICA showing Raymond-Roy class 1 embolization of the aneurysm; the asterisks show the proximal and distal markers of the NAS. AN, aneurysm; DSA, digital subtraction angiography; ICA, internal carotid artery; MCA, middle cerebral artery; NAS, Neuroform Atlas stent.

During Neuroform EZ stent deployment, when the delivery wire and microcatheter are pushed, there is more room for stent struts as well as greater cell opening for prolapse into the aneurysm orifice due to its open-cell design, such that the arterial branch from the aneurysm neck can be preserved (50). The NAS is the successor of the Neuroform EZ stent and can be used for this technique, even for small aneurysms (Figure 9).

In China, the herniation technique performed with the NAS is known as the “vault technique”. To obtain a better effect, it is necessary to select a large NAS with a diameter of 4.5 mm. However, a limitation of the technique is that the NAS is not visible on conventional digital subtraction angiography; thus, its correct positioning cannot be confirmed. In addition, this technique is useful only in the greater curvature of the artery because the struts fit the vessel wall and protect against coil migration (50).

Aside from the open-cell Neuroform stent and NAS, braided stents, such as the low-profile visualized intraluminal support (LVIS) stent (Microvention, Tustin, CA, USA), can be used to achieve a similar effect *via* the barrel or bulging technique (51, 52). The LVIS stent can expand, or barrel, to provide greater neck coverage and reduce the need to select a difficult, obtusely arising branch artery; however, because its expansion is limited to 0.2 mm beyond the unconstrained diameter, a larger LVIS stent is necessary (53). The herniation technique with the NAS and the barrel technique with closed-cell braided stents have some similarities in terms of theory.

## Rescue Stenting

Rescue stenting using the NAS is mainly used in cases of coil protrusion into the parent artery and coil migration (54–57).

### Coil Protrusion

Many factors can result in coil protrusion into the parent artery, including mismatch between the coil and aneurysm, inadequate positioning of the microcatheter in the aneurysm sac, or a wide aneurysm neck without a balloon or stent (54, 55). Coil protrusion can be categorized into the following three grades: in grade I, a loop or coil protrudes into less than half of the main lumen of the parent artery; in grade II, a coil protrudes into more than half of the main lumen of the parent artery; and in grade III, a loop protrudes into more than half of the main lumen of the parent artery (56). Coil herniation, especially grade II and III herniation, can cause turbulent flow and evoke a thromboembolic response (54).

The NAS has an open-cell structure along most of its length; therefore, it exerts a high radial force, allowing good wall apposition even in small and tortuous vessels. It can push herniated coils against the parent vessel to prevent thromboembolic events and preserve the parent artery. NAS rescue stenting is a suitable time-saving option because of the small caliber of the delivery microcatheter and the ability to deliver the NAS using the same microcatheter used for coiling. In a report by Semeraro et al., NAS rescue stenting was performed in 12 cases of coil protrusion (58). Furthermore, in a report by Shim et al., NAS rescue stenting was performed in 10 cases (54). In all of these cases, the coil protrusion was resolved.

**Figure 10:**
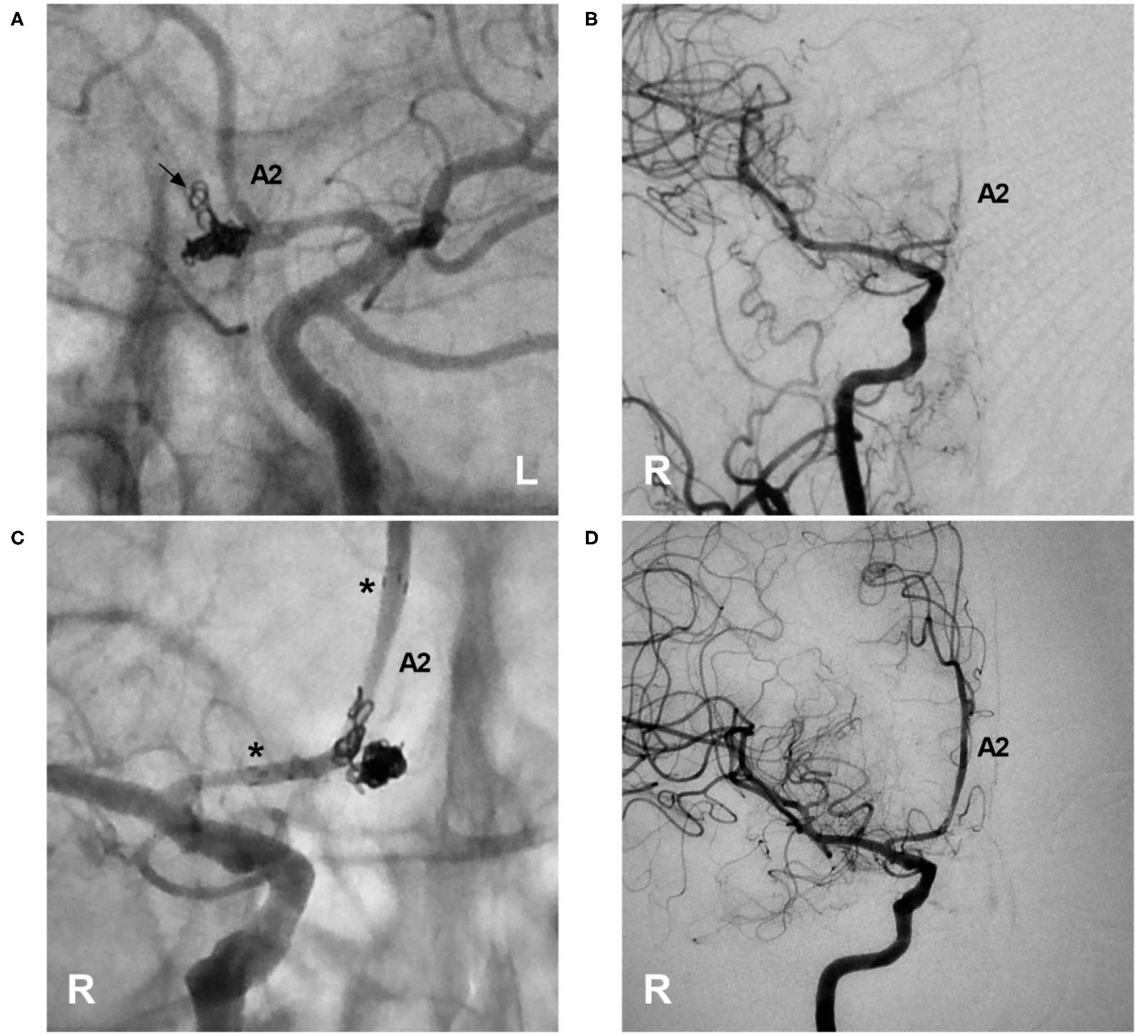
Rescue stenting with the NAS to address coil protrusion. **(A)** Unsubtracted DSA of the left ICA showing the complete coiling of an AcomA aneurysm; coil protrusion (arrow) into the contralateral artery through the AcomA was suspected. **(B)** DSA of the right ICA confirming coil protrusion and showing occlusion of the right A2 segment. **(C)** Unsubtracted DSA of the right ICA showing deployment of the NAS across the coil protrusion; the asterisks indicate the proximal and distal tips of the NAS. **(D)** DSA of the right ICA showing recanalization of the right A2 segment and restoration of normal blood flow. A2, second segment of the anterior cerebral artery; AcomA, anterior communicating artery; DSA, digital subtraction angiography; ICA, internal carotid artery; NAS, Neuroform Atlas stent; L, left; R, right.

Occasionally, when a vessel adjacent to an aneurysm is occluded by unintentional coil protrusion, NAS deployment is a good choice to restore the artery lumen (Figure 10).

### Coil Migration

Coil migration is a well-known event, occurring in 2–6% of EVT procedures (59). Recently, the rate has decreased compared with those in previous reports. For instance, in a report by Abdalkader et al., 6,071 aneurysms were treated by coiling, and coil migration occurred in 0.3% (18/6,071) of cases; a small aneurysm with an aspect ratio <1.6 and a small coil were found to be significant risk factors for coil migration (57).

Strategies for the management of coil migration include conservative medical treatment, surgical extraction, and endovascular coil retrieval (60). Distally located and asymptomatic delayed coil migration may be managed with conservative medical treatment. Acute coil migration may lead to serious ischemic complications and requires urgent treatment, especially when the migrated coil is proximally located and/or associated with vessel occlusion (59).

**Figure 11:**
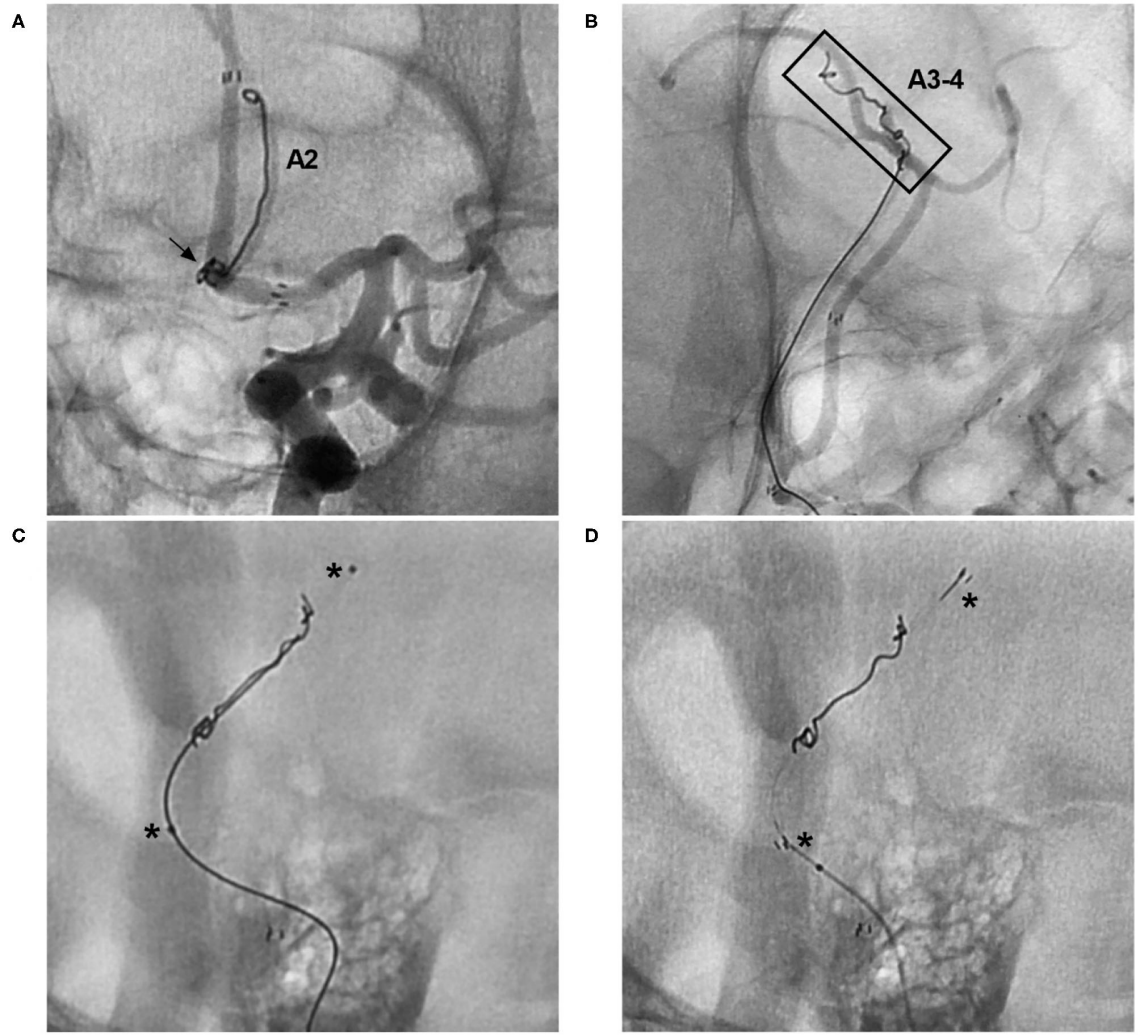
Rescue stenting with the NAS to address coil migration. **(A)** Unsubtracted DSA of the ICA showing coil protrusion from an AcomA aneurysm (arrow) into the A2 segment. **(B)** Unsubtracted DSA of the ICA showing coil protrusion from an AcomA aneurysm reaching the A3-4 segment (frame). **(C)** Unsubtracted DSA showing crossing of the coil by an XT-17 microcatheter; the asterisks indicate two markers of the microcatheter. **(D)** Unsubtracted DSA showing crossing of the coil by the NAS; the asterisks indicate the proximal and distal markers of the NAS. A2, second segment of the anterior cerebral artery; A3-4, third and fourth segments of the anterior cerebral artery; AcomA, anterior communicating artery; DSA, digital subtraction angiography; ICA, internal carotid artery; NAS, Neuroform Atlas stent.

A retrieval device and stent retriever can be used to remove a migrated coil. While the NAS cannot be used to retrieve a migrated coil, this stent has a unique advantage: it can reach very distal arteries to push the coil against the vessel wall and restore the lumen because of the small caliber of the delivery microcatheter and small, soft open-cell design (Figure 11).

## Distal Aneurysms

**Figure 12:**
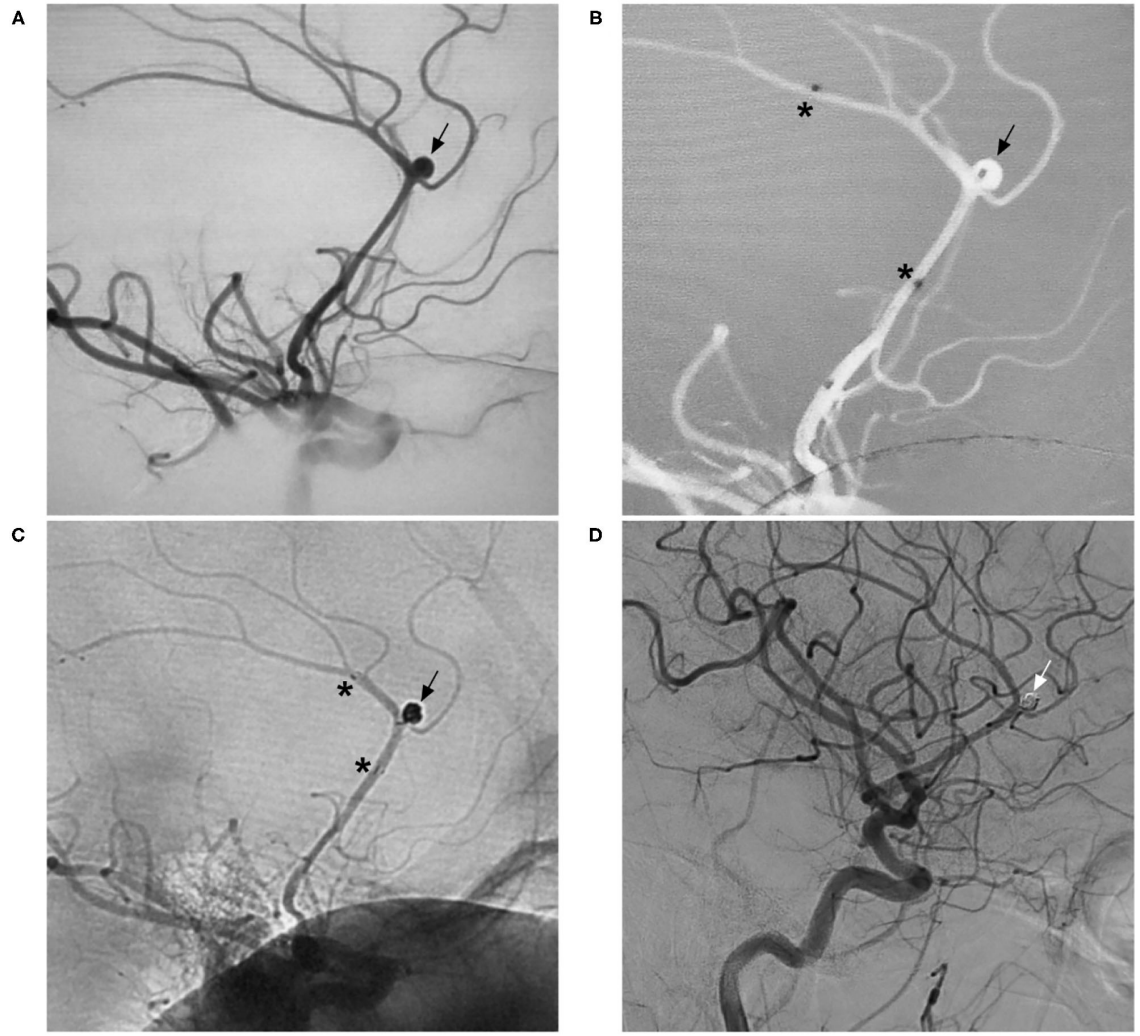
Distal aneurysm coiling assisted by the NAS. **(A)** DSA of the ICA showing an aneurysm (arrow) in the distal ACA. **(B)** Map showing the positioning of the NAS delivery microcatheter (the asterisks show the proximal and distal markers) and coil delivery microcatheter (the arrow shows the tip). **(C)** Unsubtracted DSA of the ICA showing coiling of the aneurysm (arrow) under NAS assistance (the asterisks show the proximal and distal markers). **(D)** Follow-up DSA of the ICA showing complete occlusion of the aneurysm (arrow). ACA, anterior cerebral artery; DSA, digital subtraction angiography; ICA, internal carotid artery; NAS, Neuroform Atlas stent.

The NAS is appropriate for delivery in small and/or tortuous vessels. Therefore, it can assist in the coiling of distal aneurysms. In a trial of the NAS based on an Italian multicenter registry, distal anterior cerebral artery aneurysms accounted for 8.8% (10/113) of cases (17). In a trial of NAS-assisted coiling of anterior circulation aneurysms, the NAS was used in the distal anterior cerebral artery, accounting for 2.2% of aneurysms, and in the distal middle cerebral artery, accounting for 1.6% of aneurysms (9). In a trial of NAS-assisted coiling of posterior circulation aneurysms, the NAS was used in the superior cerebellar artery and PICA, accounting for 8.6% of aneurysms (11). In EVT for distal aneurysms, two techniques can be applied. One is traditional jailing by applying two microcatheters (Figure 12). The other consists of first using a microcatheter to deploy the NAS and then traversing the stent with the microcatheter after completely covering the aneurysm to coil the aneurysm by the transcell technique; because distal aneurysms have small-caliber parent arteries, the limited space often results in difficulty during the transcell procedure (61).

## Complications of NAS Application

In three clinical trials of the NAS, the complication rate was ~5% (9–11). In a trial of NAS-assisted coiling of anterior circulation aneurysms, the rate was 4.4% (9). In a trial of NAS-assisted coiling of posterior circulation aneurysms, the rate was 4.3% (11). In the multicentric European post-market follow-up study of NAS-assisted coiling, the overall complication rate was 5.7% (10). In two systematic reviews and meta-analyses, the complication rate was ~6% (4, 14).

The complications associated with NAS application include unsuccessful NAS deployment that is completed with additional devices, acute in-stent thrombosis, chronic in-stent stenosis, and vessel perforation (7, 9, 10, 54). Dual NAS placement may have a higher but still acceptable complication rate. In Aydin et al.'s report of NAS Y-stenting, the procedural complication rate was 6.7% (29). In Ciccio et al.'s report of NAS Y- and X-stenting, the symptomatic procedural complication rate was 12.7% (26). These results are similar to those of recent meta-analyses (33, 34).

## Antiplatelet Requirements

While preoperative and postoperative dual antiplatelet therapy is necessary, no protocol has been established for the dose or duration of antiplatelet therapy that should accompany NAS placement, even in clinical trials of the NAS (9–11). In a trial of NAS-assisted coiling of anterior circulation aneurysms, patients undergoing EVT received daily oral aspirin and clopidogrel for ≥5 days, and a posttreatment dual antiplatelet regimen was maintained for at least 3 months, followed by long-term single-antiplatelet therapy (9).

In our center, nearly 500 NASs per year have been deployed to assist with aneurysm coiling, and ischemic complications have been rare intraoperatively and postoperatively. Based on our experience, due to the low metal volume and good apposition of the NAS for conformation to the vessel wall, this stent may require antiplatelet therapy at a lower dose and over a shorter period than other stents.

Pre-EVT antiplatelet requirements differ between ruptured and unruptured aneurysms. A loading dose of oral aspirin (300 mg) and clopidogrel (300 mg) can be given at least 3 h before EVT in cases of ruptured aneurysms, while a 3-day regimen of oral aspirin (100 mg/day) and clopidogrel (75 mg/day) is sufficient for unruptured aneurysms. If double NASs are planned for an unruptured aneurysm, a 7-day regimen of oral aspirin (100 mg/day) and clopidogrel (75 mg/day) is sufficient.

The appropriate post-EVT dual antiplatelet therapy differs according to the location of the aneurysm. When the NAS is deployed in aneurysms of large-diameter segments of the intracranial carotid artery, 1 month of post-EVT dual antiplatelet therapy is sufficient. When the NAS is deployed in other aneurysms of the anterior circulation and in aneurysms of the posterior circulation, dual antiplatelet therapy should be administered for up to 3 months, followed by single antiplatelet therapy for 6 months or for life. When the two NASs are deployed, dual antiplatelet therapy should be administered for 6 months, followed by single antiplatelet therapy for life.

## Summary

The NAS is compatible with low-profile microcatheters and can pass through small and highly tortuous and small vessels. According to this review and our experience, the NAS is a promising device for treating intracranial aneurysms, especially complex and distal aneurysms. The NAS can also be a powerful tool to assist in rescuing coil migration, completing dual-stent reconstruction, and coiling aneurysms *via* a transcirculation approach. The NAS may require antiplatelet therapy at a lower dose and over a shorter period than other stents. Deploying the NAS to assist in coiling aneurysms can yield good clinical outcomes and an acceptable rate of complications.

## Author Contributions

JY contributed to the conception and design of the manuscript and critically revised the manuscript. JY and KH wrote the manuscript and collected the medical records of the patients. All authors approved the final version of this manuscript.

## Conflict of Interest

The authors declare that the research was conducted in the absence of any commercial or financial relationships that could be construed as a potential conflict of interest.

## Publisher's Note

All claims expressed in this article are solely those of the authors and do not necessarily represent those of their affiliated organizations, or those of the publisher, the editors and the reviewers. Any product that may be evaluated in this article, or claim that may be made by its manufacturer, is not guaranteed or endorsed by the publisher.
